# Effect of *Amygdalus scoparia* kernel oil consumption on lipid profile of the patients with dyslipidemia: a randomized, open-label controlled clinical trial

**DOI:** 10.18632/oncotarget.18956

**Published:** 2017-07-04

**Authors:** Mohammad Javad Zibaeenezhad, Maryam Shahamat, Seyed Hamdollah Mosavat, Armin Attar, Ehsan Bahramali

**Affiliations:** ^1^ Cardiovascular Research Center, School of Medicine, Shiraz University of Medical Sciences, Shiraz, Iran; ^2^ Department of Cardiovascular Medicine, School of Medicine, Yasuj University of Medical Sciences, Yasuj, Iran; ^3^ Student Research Committee, Shiraz University of Medical Sciences, Shiraz, Iran; ^4^ Research Center for Traditional Medicine and History of Medicine, Shiraz University of Medical Sciences, Shiraz, Iran; ^5^ Cardiovascular Research Center, TAHA Clinical Trial Group, Shiraz University of Medical Sciences, Shiraz, Iran; ^6^ Noncommunicable Diseases Research Center, Fasa University of Medical Sciences, Fasa, Iran

**Keywords:** Amygdalus scoparia, dyslipidemia, nuts, nutrition, traditional medicine

## Abstract

**Background:**

Amygdalus scoparia kernel (ASK) oil is traditionally used for Hyperlipidemia. Compared to olive oil, it has higher proportion of unsaturated to saturated fatty acid besides exhibiting higher index of oxidative stability. The lipid-lowering effects of ASK oil however, has not been investigated yet. This study is the first one to evaluate such effects in patients with dyslipidemia.

**Results:**

Serum triglyceride levels significantly decreased in the intervention compared to control group (24.80 ± 51.70 vs 3.13 ± 44.80, *p*-value = 0.03). Serum total cholesterol, LDL and HDL cholesterol levels did not change significantly (*p* = 0.28 and *p* = 0.68 and *p* = 0.10 respectively).

**Materials and Methods:**

In a double arm, open-label, randomized controlled trial,101 hyperlipidemic patients were recruited. The designation of hyperlipidemia was upon meeting either of the three criteria: having serum low-density lipoprotein (LDL) cholesterol level 130–190 (mg/dl), serum triglyceride level 150–400 (mg/dl), and serum high-density lipoprotein (HDL) cholesterol level less than 50 (mg/dl) for women and 40 (mg/dl) for men. Patients who have ever been prescribed with an antihyperlipidemic medication were excluded. They were randomly assigned to intervention group, receiving the ASK oil, for 60 days and control group. Serum lipid measurements were repeated at the end of the intervention period.

**Conclusions:**

ASK oil supplementation may have a positive effect in reducing serum triglyceride level in patients with dyslipidemia without significant effect on serum cholesterol levels.

## INTRODUCTION

Cardiovascular diseases (CVD) are collectively the most important chronic diseases leading to morbidity in the world and are the leading cause of death in many countries [1]. Previous reports showed that more than 30% of the population are affected with CVD [2], and coronary artery disease (CAD) accounts for the highest mortality [3]. Among the conventional CV risk factors, dyslipidemia is a major one [4, 5] which frequently coexists with metabolic syndrome [6, 7].

Management of dyslipidemia has proven effects to decrease the CAD risk and morbidity [8]. Therapeutic strategies for dyslipidemia ranges from patient’s lifestyle modifications to drug therapy. Dietary alterations as one of the most effective components of life style modification scheme, has been reported to be largely beneficial in CAD prevention [9]. Besides, herbal dietary supplements are becoming increasingly popular among patients with dyslipidemia [10]. Although there are some clinical trials on effectiveness of these herbs and dietary supplements but still there are lack of enough investigation on many others [11, 12].

It is now well documented that nuts can improve blood lipid profile and reduces the risk of CAD and especially walnut is shown to have lipid lowering and antihypertensive effects [13–15]. Almond and its products are also among the popular dietary supplements used in the treatment of dyslipidemia and some previous studies had shown efficacy of almond on reducing plasma triglyceride, total and low density lipoprotein (LDL) cholesterol [16]. *Amygdalus scoparia*, a wild species of almond that grows in large quantities in many parts of Iran and other Middle Eastern countries, is frequently used by people for nutritional and medicinal purposes. In the only study looking for the constituents of *Amygdalus scoparia*, the chemical analysis of the oil extract by means of gas-liquid chromatography method has revealed a high proportion of unsaturated to saturated fatty acids (7.50) compared to olive oil (4.61), along with a high index of oxidative stability [17]. It had larger proportions of C18:1 and C18:2 and smaller proportions of C16:0 and C18:0 fatty acids which reveals the smaller saturated fatty acid content of *Amygdalus scoparia* oil compared to that of the olive oil. Clinically however, there is no evidence on the efficacy of consuming this almond’s species extracts for dyslipidemia. Since the high proportion of unsaturated fatty acids in nuts generally, has been documented to have a positive effect on lipid profile of patients, we aimed to evaluate the effects of *Amygdalus* scoparia kernel (ASK) oil in particular, on lipid profile of the patients with dyslipidemia in a randomized, open-label controlled clinical trial.

## RESULTS

**Figure 1:**
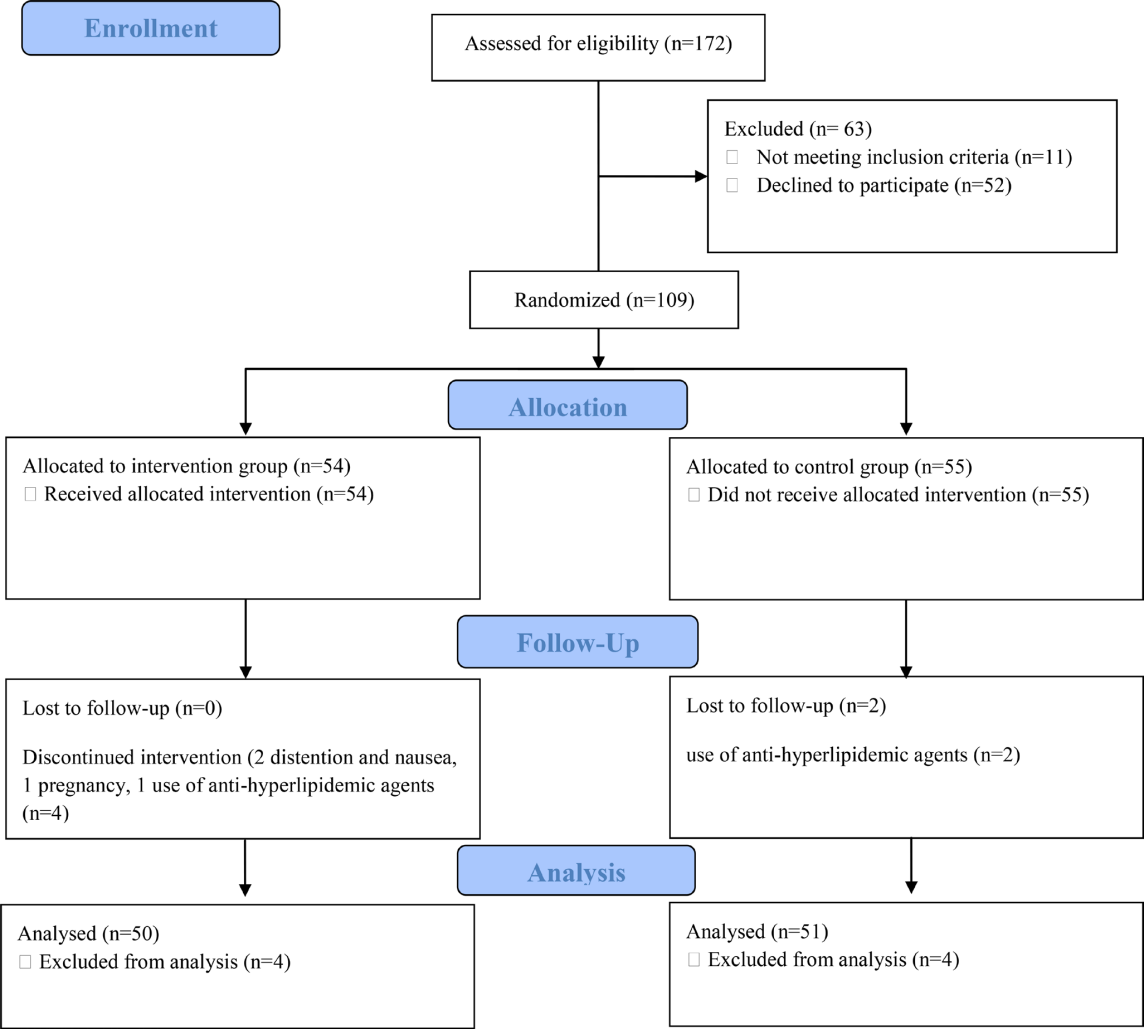
CONSORT Flow diagram of the enrolment, groups' allocation, interventions, follow up, and analysis of results

From May 2014 to April 2015, a total of 172 patients were assessed for eligibility. Among the 161 who met the inclusion criteria, 52 patients refused to participate in the trial and finally 109 patients were included in the study. Randomly 54 of them were allocated to intervention group and 55 patients were allocated to control group. Figure 1 is a flow diagram of the enrolment process, groups’ allocation, interventions, follow up, and analysis of the results. In the intervention group, two patients discontinued ASK oil because of distention and nausea and were excluded from the study. Moreover, one participant become pregnant and another one started using lipid lowering agents and were therefore excluded. Among the control group, two participants left the study and two started statins and were excluded.

The mean age of participants was 46.5 ± 11.4 and 47.2 ± 12.3 years in intervention and control groups, respectively (*p* = 0.74). The baseline clinical characteristics of the patients in both groups are shown in Table 1.

**Table 1:** Demographic data and baseline clinical measurements of the patient in intervention and control group

Basic characteristics	Intervention group (*n* = 50)	Control group (*n* = 51)	*P*-value
Mean age (years)	46.5 ± 11.4	47.2 ± 12.3	0.74
Mean body mass index (kg/m^2^)	26.9 ± 4.3	27.4 ± 2.8	0.49
Systolic blood pressure (mmHg)	123.1 ± 16.1	121.1 ± 14.5	0.49
Diastolic blood pressure (mmHg)	74.8 ± 9	73.6 ± 12	0.55

Mean serum triglyceride, total cholesterol and LDL cholesterol levels in addition to total cholesterol to HDL ratio decreased significantly in the intervention group compared to baseline, while no significant change was observed in HDL and non-HDL cholesterol levels (Table 2).

**Table 2:** Mean values (Mean ± SD) for lipids levels in intervention and control groups before and after the study

Lipid Parameters	Intervention group			*P* value*	Control group			*P* value*	Treatment effect	95% confidence interval	*P* value^†^
	Before	After	Difference		Before	After	Difference				
**Triglyceride** (mg/dL)	230.71 ± 79.94	205.91 ± 83.28	24.80 ± 51.70	0.002	183.97 ± 54.51	180.84 ± 67.27	3.13 ± 44.80	0.645	−21.66	−42.06 to −1.26	0.03
**Total cholesterol** (mg/dL)	237.51 ± 38.61	224.57 ± 41.58	12.93 ± 28.93	0.004	221.76 ± 31.36	215.02 ± 42.34	6.74 ± 26.63	0.089	−6.19	−17.58 to 5.20	0.28
**Low density lipoproteins** (mg/dL)	121.28 ± 22.88	115.28 ± 23.32	6.00 ± 16.26	0.013	119.14 ± 20.60	111.69 ± 24.72	7.44 ± 18.69	0.008	1.44	−5.57 to 8.47	0.68
**High density lipoproteins** (mg/dL)	49.54 ± 9.71	50.78 ± 9.23	−1.23 ± 6.24	0.192	54.00 ± 12.83	53.04 ± 11.51	0.95 ± 6.47	0.316	2.19	−.044 to 4.83	0.10
**Non-HDL cholestrol**	188.15 ± 33.96	173.59 ± 37.97	14.56 ± 27.27	0.122	169.60 ± 29.73	163.34 ± 40.01	6.25 ± 25.00	0.001	−8.31	−19.67 to 3.05	0.15
**Total cholesterol to HDL ratio**	4.84 ± 0.72	4.48 ± 0.73	0.35 ± 057	< 0.001	4.38 ± 0.94	4.19 ± 0.83	0.18 ± 0.67	0.080	−0.17	−0.43 to 0.09	0.21

^*^Shows the significance level for changes in means in each group (intervention and control) before and after 60 days.

^†^Shows the significance level for changes in means between the intervention and control groups after 60 days.

In the control group, the mean level of serum LDL cholesterol (119.14 ± 20.60 vs. 111.69 ± 24.72 mg/dL *p* = 0.008) and non-HDL cholesterol decreased significantly compared to baseline values. However, no significant changes were observed in serum levels of triglyceride, total cholesterol, HDL cholesterol and total cholesterol to HDL ratio in this group (Table 2).

Comparison of the changes in outcomes between intervention and control groups showed a significant reduction in level of triglyceride in participants using ASK oil compared to control group (24.80 ± 51.70 vs 3.13 ± 44.80, *p*-value = 0.03). However, there was no significant difference in other outcomes (Table 2).

### Safety and tolerability

Two patients presented with symptoms of distention and nausea by consumption of ASK oil that discontinued oil use.

## DISCUSSION

The results of this study showed that *Amygdalus scoparia* oil supplementation can affect lipid profile favorably particularly in reducing serum triglycerides. This is the first clinical trial to document the positive lipid lowering effects of ASK oil and confirms a well-known reputation of this product in Persian traditional medicine.

Although there are not previous animal or human studies on the effect of *Amygdalus scoparia* on dyslipidemia, the observed effects are in agreement with the studies on nuts oil supplementation in dyslipidemia [5, 18, 19] where they have consistently reported positive effects on lipid profile [20]. Epidemiologic and clinical trial evidence have demonstrated multiple beneficial effects of nuts and biological mechanisms underlying such effects have been suggested. Walnuts for example are shown to improve lipid profile in hyperlipidemic patients [21, 22] and its oil supplementation is reported to reduce triglycerides [23]. Jamshed and Gilani showed that almonds also, inhibit dyslipidemia in animal models through multiple pathways including inhibition of de novo cholesterol synthesis [19]. Reports on the effects of nuts on clinical outcomes are numerous. Prevention from CAD, diabetes and sudden death associated with short term feeding trials, have been attributed to rich unsaturated fatty acid constituents of nuts [24]. Some potential mechanisms for lipid lowering effects of unsaturated fatty acids involve the decrease in cholesterol absorption and synthesis and increase in excretion of neutral and acidic steroids [25]. They also promote transfer of cholesterol from plasma to tissues, alter the cholesterol-to-protein ratio in LDL and change the rates of synthesis or catabolism of individual lipoproteins [25]. It is also showed that replacement of saturated fats by unsaturated fatty acids restores LDL-receptor affinity and decreases LDL-cholesterol concentrations [24]. Furthermore, long chain polyunsaturated fatty acids have been associated with increase in expression of genes that result in resistance to cardiac ischemia [26]. They also decrease cardiomyocyte insulin resistance and improve endothelial function along with inhibition of vascular smooth muscle cell proliferation which can ameliorate atherosclerosis process and lead to plaque stability [27].

*Amygdalus scoparia* has a high content of unsaturated fatty acids [17] so that the ASK oil unsaturated to saturated fatty acid ratio is nearly twice as the olive oil. Seafood consumption which has been associated with a reduced risk of primary cardiac arrest is shown to increase cell membrane unsaturated fatty acid content so that a proportion increase from 3.3% to 5% of total fatty acids was associated with 70% reduction in the risk of primary cardiac arrest [28]. Mediterranean diet which is rich in olive oil and nuts including almonds, also brings about cell membrane structural alterations. Polyunsaturated fatty acid content of cell membrane has been only modified by Mediterranean diet plus nuts compared with a low fat diet in a randomized clinical trial [29]. The same favorable outcome can be anticipated with ASK oil as well however, this probable dual benefit remains to be addressed in another study that looks for cellular structural changes secondary to ASK oil consumption.

With the current study’s findings and regarding the oxidative stability of ASK oil [17], it can be proposed as a proper dietary substitute for other cooking oils, however longer follow up periods to investigate the long term cardiovascular effects of this herbal remedy in Persian traditional medicine is warranted for a definite conclusion.

One important limitation of this study was that despite randomization, we observed a significant change in some parameters of the lipid profile in the control group, especially LDL cholesterol. This can be the result of dietary discretion to consume less fat and subtle life style modification of participants after being told that they have been diagnosed with hyperlipidemia. Although we asked participants to continue their regular daily habits and maintain their diet consistent, a lack of dietary and physical activity documentation during the study by means of specific tools like food frequency and physical activity questionnaires, renders minor uncertainty to our interpretations. In addition, we didn’t control for the economic and social differences which might be present across the participants. Socioeconomic determinants could influence the lifestyle and dietary habits of participants at baseline and during the intervention, though randomization minimized its effects.

## MATERIALS AND METHODS

### Study design

This study is a double arm, open-label, randomized controlled clinical trial that was registered by Iranian Registry of Clinical Trials with the code: IRCT201411121525N3. The study was also approved by Local Medical Ethics Committee of Shiraz University of Medical Sciences (approval number: 7191). The final goal was to evaluate the effect of ASK oil on lipid profile of the patients with dyslipidemia.

### Participants

Men and women aged 20 to 70 years with LDL cholesterol level 130–190 (mg/dl), and/or triglyceride 150–400 (mg/dl), and/or high-density lipoprotein (HDL) cholesterol level less than 50 (mg/dl) in women and less than 40 (mg/dl) in men who were not taking lipid lowering medications were enrolled. Participants were selected among those referred to Shiraz Heart Health House as volunteers. They all received instructions by a cardiologist and signed an informed consent form of participation in the study.

Exclusion criteria were history of ischemic heart disease, renal, liver and thyroid disease, history of diabetes mellitus, history of food allergy especially to soya, peanuts and walnuts, history of asthma or atopic dermatitis, pregnancy and lactation, taking oral contraceptive pills and alcohol consumption. During the intervention period, we asked participants not to use any other lipid lowering drugs or alter their routine dietary and exercise habits. Those hospitalized during the study or developing diarrhea more than 5 episodes per day were subsequently excluded.

### Intervention

Patients were randomly assigned to receive either ASK oil, as the intervention group, or without intervention as the control group in a period of two months. They were asked to refrain from consumption of *Amygdalus scoparia*-containing foods, vitamin supplements and herbal preparations two weeks before starting the study (as washout period). Enrolled patients received cans containing one liter of cold pressed ASK oil, produced by Mashhad plants oil industrial unit, and were asked to consume 10 cc per day orally for 60 days. Chemical analysis of the extracted oil was as follow: oleic acid, 72.7%; palmitic acid, 10.4%; linoleic acid, 10.3%; stearic acid, 6.1%; palmeolytic acid, 0.3%; gadoleic acid, 0.1% and arachidonic acid, 0.1%. The overall lipid content was 39.1 ± 1%, protein content, 6 ± 1% and water 37.6 ± 0.2%. In order to reduce errors in consumed oil dose, the disposable plastic cups with capacity of 10 cc were provided for the patients and they were asked to take a cup of the ASK oil in daily food.

### Randomization

Randomization was done using a computer-based random digit generator based on the registration number of the patients (on the order of referral). Only the statisticians were blind to the allocation of the patients.

### Outcome

Serum lipid profile including total, LDL, HDL, non-HDL cholesterol levels, total cholesterol to HDL ratio, and triglyceride levels were determined at the baseline and 60 days after the intervention.

### Measurements

The serum triglyceride was measured by GPO-PAP method providing a normal upper limit of 200 mg/dL (2.3 mmol/L). The total cholesterol was also checked by CHOD/PAP technique, which provided an upper limit normal value of 220 mg/dL (5.6 mmol/L). The HDL cholesterol was measured by dextran magnesium sulfate. The LDL cholesterol was derived according to the following formula: LDL = total cholesterol – (HDL +TG/5) [30].

### Statistical analysis

The sample size was calculated by considering significance level of 5% and 95% power and by considering probable 10% drop-out rate; the sample size was calculated to be 55 patients in each group. Gathered data were analyzed using Statistical Package for the Social Sciences (SPSS) software (Version: 15). Statistics are represented by mean ± standard deviation (SD). Chi square paired, and independent Samples *t* test were conducted for data analysis as the gathered data were normally distributed with equal variances. The significance level less than 0.05 was considered in all cases.

## CONCLUSIONS

It can be concluded that ASK oil supplementation may have positive effects on control of triglyceride in patients with dyslipidemia, and may be beneficial as a supplement or dietary intervention for patients with hypertriglyceridemia.

## Abbreviations

CVD: cardiovascular disease
CAD: coronary artery disease
ASK: *Amygdalus scoparia* kernel
LDL: low density lipoprotein
HDL: high density lipoprotein
